# The early economic evaluation of novel biomarkers to accelerate their translation into clinical applications

**DOI:** 10.1186/s12962-018-0105-z

**Published:** 2018-06-18

**Authors:** Gimon de Graaf, Douwe Postmus, Jan Westerink, Erik Buskens

**Affiliations:** 1 Department of Epidemiology, University of Groningen, University Medical Center Groningen, PO Box 30.001, 9700 RB Groningen, The Netherlands; 2 Department of Vascular Medicine, University Medical Center Utrecht, PO Box 85.500, 3508 GA Utrecht, The Netherlands

**Keywords:** Early health technology assessment, Translational research, Headroom analysis, Biomarkers, Cardiovascular disease risk

## Abstract

**Background:**

Translating prognostic and diagnostic biomarker candidates into clinical applications takes time, is very costly, and many candidates fail. It is therefore crucial to be able to select those biomarker candidates that have the highest chance of successfully being adopted in the clinic. This requires an early estimate of the potential clinical impact and commercial value. In this paper, we aim to demonstratively evaluate a set of novel biomarkers in terms of clinical impact and commercial value, using occurrence of cardiovascular disease (CVD) in type-2 diabetes (DM2) patients as a case study.

**Methods:**

We defined a clinical application for the novel biomarkers, and subsequently used data from a large cohort study in The Netherlands in a modeling exercise to assess the potential clinical impact and headroom for the biomarkers.

**Results:**

The most likely application of the biomarkers would be to identify DM2 patients with a low CVD risk and subsequently withhold statin treatment. As a result, one additional CVD event in every 75 patients may be expected. The expected downstream savings resulted in a headroom for a point-of-care device ranging from €119.09 at a willingness to accept of €0 for one additional CVD event, to €0 at a willingness to accept of €15,614 or more.

**Conclusion:**

It is feasible to evaluate novel biomarkers on outcomes directly relevant to technological development and clinical adoption. Importantly, this may be attained at the same point in time and using the same data as used for the evaluation of association with disease and predictive power.

## Background

Within the current paradigm of personalized medicine or precision medicine, many research efforts are aimed at identifying novel biomarkers [1–3]. Although the expectations of improved clinical practice through better patient characterization remain high, it has long been recognized that the vast amount of biomarker research fails to live up to these expectations [4–10]. The fact that so few biomarkers are successfully translated from scientific discovery to clinical application entails a loss in health potential for patients and society. Moreover, resources from public and private investors allocated to research, development, and evaluation with the aim to improve patient outcomes appear wasted.

Biomarker discovery research has produced a vast body of literature on the association between biomarker and disease or outcome, and their diagnostic or prognostic performance (i.e. discrimination or reclassification) [3, 11]. While this is often regarded as the end-point of discovery research, it is only the start of the translational research phase. Herein, a candidate biomarker is developed into a diagnostic or prognostic technology and evidence required for its adoption in the clinic is generated [8, 12–15]. Akin to clinical trials for pharmaceuticals, translational research is a long and complex trajectory requiring large financial investments, and will result in the rejection of a number of biomarker candidates [13]. Expert estimates of the costs of developing and commercializing a new biomarker based diagnostic technology exceed $100M [16]. As a result, the large number of candidate biomarkers that could be developed into clinical applications far exceeds the resources available to do so. It is therefore of great importance to identify those candidate biomarkers that have the highest chance to succeed as a commercial product. This requires an estimate of their potential clinical value and cost-effectiveness [11, 15, 17]. Unfortunately, currently employed methods for early biomarker evaluation provide little insight into clinical value [8, 9]. On the other hand, proposed methods for the assessment of clinical value such as (early) health economic modeling are too extensive to be applied for the selection of biomarker candidates [13–15, 17].

The PREdiction and early diagnosis of DIabetes and diabetes-related Cardiovascular Complications (PREDICCt) project of the Center for Translational Molecular Medicine (CTMM) was initiated to enhance the possibilities for prevention of DM2 and associated complications through the development of molecular diagnostics and molecular imaging of novel biomarkers [18]. Its research efforts identified three novel biomarkers that were associated with incident CVD in DM2 patients: NT-proBNP, MMP-3, and Osteopontin. The association of these biomarkers with CVD incidence, as well as their predictive power within a prediction model have been described previously [19]. Whether further investments in translational research to develop diagnostic technologies based on these biomarkers is warranted has yet to be determined.

In this paper, we aim to demonstrate an evaluation framework for the assessment of novel biomarkers on clinical impact and commercial value (headroom). Such an assessment can be used to support the selection of biomarker candidates for further development and R&D investment decisions during development. We claim that this may be achieved at the same point in time and using the same data as used for the evaluation of predictive power or technical accuracy (i.e. data often available from discovery research). The CTMM PREDICCt project is used as a real-life case study to illustrate our framework. In our framework, we first define the application of the PREDICCt biomarkers in the clinical pathway and subsequently estimate the headroom of the markers in this application.

## Clinical application definition

Numerous publications on the translation of biomarkers stress the importance of defining a clinical application early in the discovery and development process [10, 14, 20]. This is because the value of any diagnostic or prognostic test depends on the setting in which it is applied, and the decision it is used to support. For many published biomarkers no clinical application has been specified, or this has been defined so broadly that it cannot possibly be used to determine their potential (cost-)effectiveness or commercial value. In our case study project, two very broad possible applications of the discovered biomarkers had been proposed. The first was to identify low-risk DM2 patients for whom treatment could be postponed, the second was to identify high risk DM2 patients for whom treatment could be initiated or intensified [19]. With respect to the economic value of the biomarkers it has been proposed that an individual patient risk-based approach has the apparent potential to allocate treatment resources more efficiently and effectively [19].

To define a sufficiently detailed clinical application for the biomarkers, we gathered input from two clinical experts: an internist specialized in vascular medicine (third author on this publication), and the resident cardiologist that authored the publication describing the predictive power and possible clinical application of the biomarkers [19]. Under current international guidelines, DM2 patients are regarded as a high risk group for which the prescription of statins is advised [21–24]. In terms of risk, the so called high risk-group is defined by a 10-year CVD risk of 10% or higher. Recent studies indicated that there is a wide range of risk among the DM2 patient population [25, 26]. Consequently, for part of the DM2 patient population the 10-year risk will likely fall below 10%, in which case these patients could be considered to be over-treated under current guidelines. This could potentially be remedied by using a more accurate risk prediction based on the newly discovered biomarkers. The second application of the PREDICCt biomarkers—to identify high risk patients and initiate or intensify treatment—is less likely to have a substantial clinical impact, due to the current clinical practice of CVD risk management in DM2 patients. As DM2 patients already fall in the highest risk category according to most guidelines, and given the limited options available for more intensive treatment, using the biomarkers as a risk stratification tool to select very high risk patients for intensified treatment is not a viable option. Apart from intensifying preventive treatment, high risk patients could also be screened for prevalent asymptomatic CVD. However, current guidelines clearly recommend against this practice, as it does not improve outcomes in patients that already receive preventive treatment [24].

## Headroom analysis

In this section, we aim to evaluate the clinical impact and headroom of a risk stratification tool based on the three biomarkers identified in the PREDICCt project when used to identify patients at low risk for CVD (10 year risk < 10%) and subsequently withhold statin treatment in these patients. The headroom of a new technology is the maximum net incremental cost for which its intended clinical application is still likely to be cost-effective [27]. We conducted a model-based evaluation using data from a large cohort study in The Netherlands. First, we developed a prediction model comprising the risk factors of the UKPDS risk engine [28] and the three novel biomarkers. Then, we estimated the impact of withholding treatment in those that fell below the risk cut-off using published data on the effectiveness of statins. Clinical impact was defined as the number of treatments withheld per additional CVD case. The headroom of the risk stratification tool was calculated for different levels of willingness to accept for one additional CVD event in the target population. The willingness to accept is the minimum monetary amount that the healthcare payer must save or receive in order to be willing to forgo a certain health benefit. As the current status quo is to provide the intervention to all patients, the new technology leads to reduced health benefits at lower costs. Thus, willingness to accept is an appropriate measure of preference, rather than the more ubiquitous willingness to pay, which applies when an additional benefit can be obtained at an additional cost.

### Study population

We used patient level data from the Secondary Manifestations of ARTerial disease (SMART) study, a prospective cohort from The Netherlands. This study included patients that were referred to hospital with either manifest artherosclerotic disease or for the management of cardiovascular risk factors, such as hypertension, hyperlipidaemia, and DM2. A detailed description of the study design has been published previously [29].

For the purpose of the current study, we selected patients with DM2 that had at least 5 years of follow-up and no prior history of CVD at the time of inclusion (n = 389). DM2 was defined as a referral diagnosis of DM2, self-reported DM2, the use of glucose-lowering agents, or a plasma glucose concentration of ≥ 7.0 mmol/L at baseline combined with the initiation of glucose-lowering treatment within 1 year after inclusion. Patients were considered to have a prior history of CVD when their medical records stated cerebrovascular disease (transient ischemic attack, cerebral infarction, cerebrovascular ischemia, amaurosis fugax, or retinal arterial occlusion), peripheral vascular disease, coronary artery disease, or an abdominal aortic aneurysm. The characteristics of the study population included in our analysis are shown in Table 1.

**Table 1:** Study population characteristics

Parameter	Baseline value
Age [years, mean (SD)]	54.8 (11.0)
Female sex (%)	39.8
Age at diagnosis of type-2 diabetes [years, mean (SD)]	49.8 (11.6)
Currently smoking (%)	24.9
HbA1c [%, median (IQR)]	7.4 (6.6–8.6)
Systolic blood pressure [mmHg, mean(SD)]	145 (21)
Total cholesterol/HDL cholesterol ratio [median (IQR)]	4.6 (3.7–6.1)
NT-proBNP [pg/mL, median (IQR)]	92 (44–216)
MMP-3 [ng/mL, median (IQR)]	12.4 (8.1–17.3)
Osteopontin [ng/ml, median (IQR)]	17.0 (13.3–21.9)

Patient characteristics of the 389 patients without prior cardiovascular disease history in the SMART cohort

*SD* standard deviation, *IQR* interquartile range

### Risk assessment

The 10-year CVD risk (defined as the occurrence of myocardial infarction, stroke or vascular death) for each patient in the study population was calculated using an internally developed risk prediction model based on the Fine and Gray methodology [30]. This model consisted of the risk factors in the UKPDS risk engine (age at diagnosis of DM2, sex, current smoking, HbA1c, systolic blood pressure, and the total cholesterol/HDL cholesterol ratio), and the three novel biomarkers. Missing values on these predictor variables in our dataset were dealt with using multiple imputation using the R-library MICE [31]. CVD risk was then computed by taking the average of the risk values predicted from each of the imputed datasets.

### Effectiveness gap

We assumed that withholding statin treatment only has an impact on the incidence of CVD events and not on the non-CVD death rate. To estimate the clinical impact of this change in treatment policy, we fitted a competing risks model predicting the 10-year incidence of CVD events to the low-risk group. The model estimated cause-specific hazards for having a CVD event and for non-CVD death. These hazards were assumed to have a proportional hazard structure described by a Weibull distribution, and are described as follows:

$$h_{CVD} \left( t \right) = \left( {\upalpha_{c} /\upbeta_{c} } \right)\left( {t/\upbeta_{c} } \right)^{{\left( {\upalpha_{c} - 1} \right)}} HR_{notreatment}$$

and

$$h_{nonCVDdeath} (t) = (\upalpha_{d} /\upbeta_{d} )(t/\upbeta_{d} )^{{(\upalpha_{d} - 1)}}$$

where α_c_ (0.098) and β_c_ (4.879) are the shape and scale parameter of the Weibull distribution for CVD events, respectively, and α_d_ (0.362) and β_d_ (4.348) the shape and scale parameter of the Weibull distribution for non-CVD death, respectively. Lastly, *HR*_*no treatment*_ is the hazard ratio for the effect of withholding treatment. A large trial on the effects of statins in DM2 patients reported a hazard ratio of 0.76 [32], and in a meta-analysis of 14 randomized trials a relative risk of 0.79 per mmol/L reduction in LDL cholesterol was found [33]. We therefore assumed that the effect of withholding statin treatment in our target population would lead to a hazard ratio of 1.25 for CVD events. The effectiveness gap was defined as the increase in 10-year CVD incidence resulting from withholding statin treatment in the low-risk group. For each treatment strategy (prescribing statins and withholding statins), these cumulative incidences were calculated as

$$I_{CVD} \left( t \right) = \mathop \int \limits_{0}^{t} h_{CVD} \left( s \right)S\left( s \right)ds$$

where

$$S(t) = \exp \left[ { - \mathop \int \limits_{0}^{t} h_{CVD} (s)ds - \mathop \int \limits_{0}^{t} h_{nonCVDdeath} (s)ds} \right]$$

is the overall survival function.

### Headroom

The costs of statin treatment were estimated to be €0.06 per day based on the average cost of simvastatin 40 mg in The Netherlands [34]. As DM2 patients will have periodic checks with their general practitioner, as well as other prescription medication, costs for physician visits and prescription filling by pharmacies were assumed not to change when withholding statin treatment. The headroom of the point-of-care device was expressed as a function of the willingness to accept for one additional CVD event:

$$H(WTA) = f_{LR} (C_{T} - \Delta I_{CVD} \cdot WTA)$$

in which *f*_*LR*_ is the fraction of patients in the DM2 population with a CVD risk below 10%, Δ*I*_*CVD*_ is the change in CVD incidence as a result of withholding statin treatment, *WTA* is the willingness to accept for one additional CVD event, and *C*_*T*_ is the average per patient cost of statin treatment over the study horizon of 10 years. This was based on the average time patients DM2 patients are alive and did not experience a CVD event in our competing risk model, and defined as:

$$C_{T} = 365.25 \cdot {\sf C}\!\!\!\!\raise.8pt\hbox{=}0.06\left( {\mathop \int \limits_{0}^{{10}} t[h_{{CVD}} (t) + h_{{nonCVDdeath}} (t)]S(t)dt + 10 \cdot S(10)} \right)$$

This willingness to accept was varied between €0 and the level at which the resulting headroom would be €0.

### Sensitivity analysis

Apart from the willingness to accept, which was varied in the base case analysis, the headroom is to a large extend determined by the cost of treatment *C*_*T*_ and the effects of withholding statin treatment on CVD incidence Δ*I*_*CVD*_. We therefore assessed the impact on the headroom of changes in the per diem cost of statin treatment and the hazard ratio for the effect of withholding statin treatment in the low-risk group. Per diem costs of statin treatment were €0.06 in the base case and were varied by 25% in the sensitivity analysis (€0.045 and €0.075). We assessed two alternative scenarios for the effects of withholding stating treatment in the low-risk group. First, we assumed that the relative effectiveness of statin treatment is related to baseline CVD risk, meaning that low-risk patients have a lower relative risk reduction as a result of statin treatment. This was implemented by using a hazard ratio for the effect of withholding statin treatment of 1.10, as opposed to 1.25 in the base case. In the second scenario we based the effects of statin treatment on a different study, which found a hazard ratio of 0.63 for the effect on CVD incidence in DM2 patients [35]. This was implemented by using a hazard ratio for withholding treatment in the low-risk group of 1.58.

### Results

**Fig. 1:**
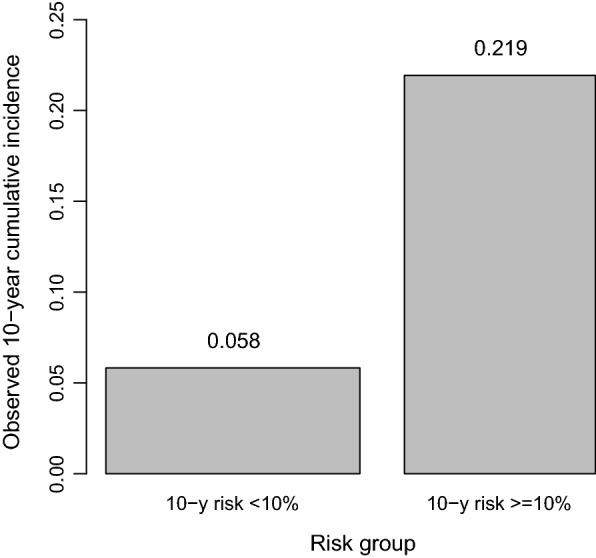
Difference in observed 10-year cumulative cardiovascular disease (CVD) incidence (bar height) and group size (bar width) between the low risk group (estimated 10-year CVD incidence < 10%) and the high risk group (estimated 10-year CVD incidence > = 10%) based on the risk prediction model consisting of age at diagnosis of DM2, sex, current smoking, HbA1c, systolic blood pressure, and the total cholesterol/HDL cholesterol ratio, NT-proBNP, MMP-3, and Osteopontin

The low-risk group (10-year CVD risk < 10%) thus identified consisted of 57.1% of the study population (Fig. 1). A large difference in the observed 10-year incidence was found between the two risk groups, indicating that the risk assessment model had a high predictive power (Fig. 1).

**Fig. 2:**
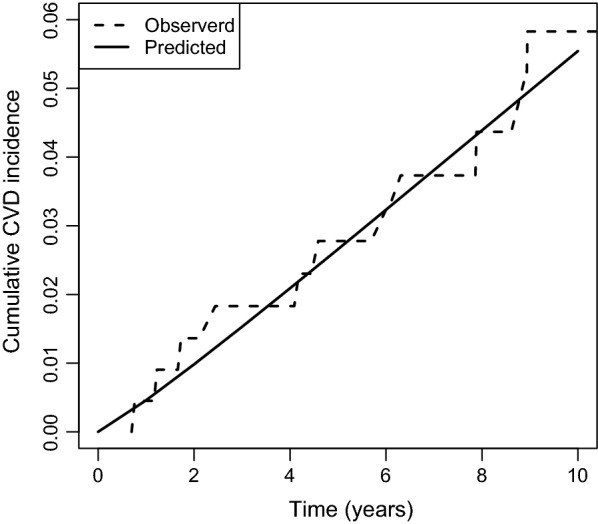
Comparison between the cumulative incidence of cardiovascular disease as predicted by the competing risk model and as observed in the SMART cohort

The predicted and observed 10-year CVD incidences are shown in Fig. 2. Withholding treatment in the low-risk group increased the predicted cumulative CVD incidence at 10 years by approximately 0.0133. This means that withholding treatment will lead to one additional CVD event in every 75 patients.

The average duration of treatment in the high risk group was estimated to be 9.52 years. This led to an estimated total average treatment cost over 10 years of €208.67. The headroom of a point-of-care device using the novel biomarkers was found to be €119.09 at a willingness to accept of €0 (that is, no savings or monetary gain would be required to accept an additional CVD event). The headroom became less than €0 when the willingness to accept for one additional CVD event exceeded €15,614 (that is, an additional CVD event is accepted when a cost saving of more than €15,614 is realized).

The results of the sensitivity analysis are shown in Table 2. Varying the treatment effect of statins did not have an impact on the maximum headroom but did impact the willingness to accept level at which the headroom becomes €0 (which increased when statin effects were less). Changes in the cost of statin treatment were reflected in the total cost of treatment and thereby had an impact on the maximum headroom (higher statin costs led to a higher headroom).

**Table 2:** Results of the sensitivity analysis

Outcome	Base case	Lesser effect of statins (HR 1.10)	Larger effect of statins (HR 1.58)	Statin cost + 25%	Statin cost − 25%
Additional CVD incidence	0.0134	0.0054	0.0307	0.0134	0.0134
Number needed to withhold	75	186	33	75	75
Total average cost of treatment	€208.67	€208.67	€208.67	€260.83	€156.50
Headroom at WTA = €0	€119.09	€119.09	€119.09	€148.86	€89.31
WTA at which headroom = €0	€15,614	€38,867	€6795	€19,518	€11,711

*HR* hazard ratio for the effect of withholding statin treatment on cardiovascular disease, *CVD* cardiovascular disease, number needed to withhold = withholding treatment in this number of patients leads one additional cardiovascular disease event

## Discussion

In this study, we demonstrated that an early assessment of the clinical impact and commercial value (headroom) of novel biomarkers can be performed at the same time and using the same data as used to determine predictive power and accuracy. We used a case study of biomarkers for additional CVD risk stratification in DM2 patients, more specifically a setting where such biomarkers would be used as a prognostic test to inform the decision on withholding statin treatment from low-risk patients. We found that withholding statin treatment in DM2 patients with a 10 year CVD risk of < 10% lead to an additional CVD event in every 75 patients for which treatment would be withheld. Furthermore, we found the headroom to be €119.09 in the optimal scenario from the industry perspective (that is, when no savings would be required in order to accept an additional CVD event). The headroom reduced to €0 when the willingness to accept would be €15,614 or more. When a larger cost saving is demanded for an additional CVD case (that is, there is a higher willingness to accept), a smaller part of the costs saved by withholding treatment is available to pay for the biomarker test. Headroom thus decreases as the willingness to accept increases. The willingness to accept at which the headroom is reduced to €0 was sensitive to changes in both the effect of statin treatment in the low-risk group, as well as the cost of statin treatment (lesser treatment effect and higher statin cost led to a higher willingness to accept at which headroom is €0). The maximum headroom was only sensitive to the cost of statin treatment (increased cost of statins led to a higher maximum headroom).

Our study is the first that estimates the clinical impact and commercial value of biomarkers for the estimation of CVD risk in DM2 patients, and one of the first to perform such an analysis for a biomarker technology before it is actually developed. A large body of literature exists demonstrating the predictive power and strength of association between biomarker and disease for many different types of biomarkers. Based on such results, there is often a positive and hopeful attitude towards novel biomarkers. These outcome measures, however, have little relation to the clinical, commercial, or economic value of a biomarker technology [11, 17]. Notably, it is not uncommon for a biomarker to be developed without a clear clinical implementation in mind. Without a clinical application definition, any assessment of clinical value or cost-effectiveness is impossible. Such evidence is crucial for the adoption of a new biomarker technology in the clinic and by extension thereof its commercial success. As a result, many novel biomarkers fail to deliver on the high hopes that have been placed on them, and represent a waste of public and private research funds. Existing methods for the economic evaluation of biomarkers (and other healthcare innovations) such as early health economic modeling require more data, are computationally more complex, and as a result demand more time and financial resources to implement [15, 36]. Assessing multiple biomarker candidates, each with multiple possible applications, is often not feasible using those methods. Our less resource-demanding method employing data from biomarker discovery research and published literature in a computationally uncomplicated approach can provide relevant support in decision making.

The methods we employ are not completely novel. A number of methodological studies have dealt with the issue of biomarker assessment, some of which focus on the statistical aspects of such an assessment [37–40], while others describe assessment in a broader scope, including decisions on area of application and current care comparators [27, 41, 42]. Our main goal was to demonstrate the applicability of such methods in a real-life setting of biomedical development. Likewise, a few recent studies demonstrated the potential for using health economic modeling as an alternative for RCTs to generate evidence on the cost-effectiveness of diagnostic tests [43, 44]. In several ways these studies have used an approach similar to ours. The main difference being that our method is aimed at an earlier stage of development—immediately after discovery—where most biomarkers are falling out of the translational process. It thereby aims to primarily inform decisions on the direction of development and investment, rather than adoption in the clinic.

Our case-study outcomes are difficult to compare to outcomes of other studies. Most economic evaluations use cost per quality adjusted life-year (QALY) as their primary outcome and determine cost-effectiveness by specifying a willingness to pay for an additional QALY. Accurately estimating the loss of QALYs as a result of withholding treatment would require a disease progression model, which is beyond the scope of this showcase research. Moreover, the applicability of QALYs as an outcome measure in modeling studies for diagnostic test has previously been questioned [17]. A further issue regarding comparability with previous research is the fact that the willingness to accept is a concept not often encountered in health economic evaluations. A threshold for willingness to accept an additional CVD event has never been specified. However, even in the absence of a relevant threshold the outcomes of our method can be informative for R&D and investment decisions. When a large headroom exists even when extremely unfavorable (i.e. low in the case of willingness to pay, high in the case of willingness to accept) threshold values are used in the analysis, further investments in the development of the new technology are certainly warranted from an economic perspective. When no or a very small headroom exists when favorable threshold levels are used, it is unlikely that the new technology will ever be cost-effective when used in the evaluated application, and therefore it would not be wise to invest in further development. By this token, due to the high costs and burden associated with cardiovascular events such as myocardial infarction and stroke, it would appear unlikely that the willingness to accept for an additional CVD case will be sufficiently low to ever make a risk stratification tool in DM2 patients like the one analyzed in our case study a viable strategy. A threshold defined in willingness to accept is rare because most new interventions provide increased health outcomes at an additional cost. However, as many societies are increasingly concerned by the sustainability of healthcare expenditures, we believe that it will become increasingly important to be able to express the willingness to forgo health benefits in return for cost reductions. These limitations notwithstanding, we believe that we have demonstrated that without using other evidence than datasets used for biomarker discovery and published literature, it is possible to go beyond the usual evaluation of biomarkers on association with disease and predictive power and additionally give an insight in potential clinical impact and commercial value.

## Abbreviations

CVD: cardiovascular disease
CTMM: Center for Translational Molecular Medicine
DM2: type-2 diabetes mellitus
UKPDS: The United Kingdom Prospective Diabetes Study
QALY: quality-adjusted life year
WTA: willingness to accept
